# Electrospun Collagen Scaffold Bio-Functionalized with Recombinant ICOS-Fc: An Advanced Approach to Promote Bone Remodelling

**DOI:** 10.3390/polym14183780

**Published:** 2022-09-09

**Authors:** Priscila Melo, Giorgia Montalbano, Elena Boggio, Casimiro Luca Gigliotti, Chiara Dianzani, Umberto Dianzani, Chiara Vitale-Brovarone, Sonia Fiorilli

**Affiliations:** 1 Department of Applied Sciences and Technologies, Politecnico di Torino, Corso Duca Degli Abruzzi 24, 10129 Torino, Italy; 2 School of Engineering, Newcastle University, Newcastle Upon Tyne NE1 7RU, UK; 3 NOVAICOS s.r.l.s, Via Amico Canobio 4/6, 28100 Novara, Italy; 4 Department of Health Sciences, Università del Piemonte Orientale, Via Solaroli 17, 28100 Novara, Italy; 5 Department of Drug Science and Technology, Università degli Studi di Torino, Via Pietro Giuria 9, 10125 Torino, Italy

**Keywords:** type I collagen, electrospinning, ICOS-Fc, cell migration, bone remodelling, osteoporosis

## Abstract

The treatment of osteoporotic fractures is a severe clinical issue, especially in cases where low support is provided, e.g., pelvis. New treatments aim to stimulate bone formation in compromised scenarios by using multifunctional biomaterials combined with biofabrication techniques to produce 3D structures (scaffolds) that can support bone formation. Bone’s extracellular matrix (ECM) is mainly composed of type I collagen, making this material highly desirable in bone tissue engineering applications, and its bioactivity can be improved by incorporating specific biomolecules. In this work, type I collagen membranes were produced by electrospinning showing a fibre diameter below 200 nm. An optimized one-step strategy allowed to simultaneously crosslink the electrospun membranes and bind ICOS-Fc, a biomolecule able to reversibly inhibit osteoclast activity. The post-treatment did not alter the ECM-like nanostructure of the meshes and the physicochemical properties of collagen. UV-Vis and TGA analyses confirmed both crosslinking and grafting of ICOS-Fc onto the collagen fibres. The preservation of the biological activity of grafted ICOS-Fc was evidenced by the ability to affect the migratory activity of ICOSL-positive cells. The combination of ICOS-Fc with electrospun collagen represents a promising strategy to design multifunctional devices able to boost bone regeneration in osteoporotic fractures.

## 1. Introduction

Bone injuries and unsuccessful fracture healing represent one of the most critical clinical burdens nowadays, often caused by pathological conditions, such as osteoporosis (OP), where the dynamic balance between the activity of bone-forming cells, osteoblasts (Ob), and resorptive ones, osteoclasts (Oc), is severely compromised [1,2]. Due to the increase in the ageing population, and the limitations associated with the currently available pharmacological treatments, the total number of people affected by impaired bone healing is predicted to sensibly grow in the coming future [3]. This unmet clinical need results in the urgency of ad hoc medical devices and personalized treatments, not only capable of stimulating bone tissue regeneration in elderly people, but also specifically adapted for the fracture type, the anatomical location, and the specific clinical requirements [4]. To face this challenge, in the field of bone tissue engineering (BTE), the combination of smart biomaterials, biofabrication technologies, and specific biological cues is under investigation to develop multifunctional 3D scaffolds capable of supporting cell proliferation, cell guidance, and to promote the restoration of the tissue’s innate natural balance [5]. In this frame, the processing of biopolymers able to induce osteogenesis, such as silk fibroin [6] and collagen [7], is widely reported for manufacturing fibrous scaffolds mimicking both the composition and the architecture of bone extracellular matrix (ECM). To this purpose, electrospinning (ESP) technologies represent one of the most exploited tools to produce biomimetic nanofibrous constructs, with fibre diameters ranging from a few microns to less than 100 nm. Moreover, the resulting electrospun matrices are normally characterized by high flexibility and large exposed surface area which makes them particularly suitable for functionalization with bioactive molecules [8].

Since collagen type I is the main component of bone ECM, its use alone, or in combination with other natural or synthetic polymers, has been widely reported for ESP of scaffolds able to promote cell adhesion and proliferation [6,9,10,11]. Despite the excellent biocompatibility and bioactivity of collagen, its processing with electrospinning technologies has frequently been associated with partial denaturation of the protein, mainly caused by the solvents and process parameters used. In addition, the poor stability in aqueous media and the fast degradation kinetics of collagen require an adequate chemical crosslinking of the scaffolds often compromises the original nanofibrous structure obtained by electrospinning, as well as the final biocompatibility [12,13,14].

Although the inclusion of osteoconductive inorganic phases, such as nanohydroxyapatite [15], or mesoporous bioactive glasses (MBGs) [16] has often been chosen as an effective strategy to improve both stability and multifunctionality of collagen-based constructs, an alternative, or complementary method, is represented by the functionalization of the fibrous meshes with specific biological cues. This can be achieved through the encapsulation or the direct covalent binding of bioactive molecules to obtain specific cell stimulation [17]. Thanks to their large exposed and accessible surface area the electrospun matrices, made of either natural or synthetic polymers, have been functionalized with growth factors and stimulatory chemicals (bone morphogenic proteins [18] and osteogenic factors [19]), to induce cell differentiation and stimulate regeneration [20], when the physiological bone remodelling is compromised or delayed (i.e., osteoporosis/osteopenia, autoimmune diseases, and bone tumours).

With this perspective, the recombinant biomolecule ICOS-Fc, patented by the authors (WO/2016/189428), successfully proved to be active on bone resorption by reversibly inhibit osteoclast activity and has consequently emerged as a powerful therapeutic approach to treat osteolytic diseases. Indeed, this recombinant biomolecule is able to bind the surface receptor (ICOSL) expressed by several cell types, including osteoclasts, and, consequently, to substantially affect their activity [17,21,22]. Accordingly, in vitro and in vivo findings have demonstrated that ICOS-Fc prevents differentiation and bone erosive activity of osteoclasts and the development of osteoporosis (OP) in mice [21].

In this work, inspired by the breakthrough of these results, the authors aimed at the design of a bioactive collagen-based device biofunctionalized with ICOS-Fc molecule, potentially intended for the stimulation of physiological bone regeneration in compromised clinical situations. In addition, the peculiar flexibility provided by the electrospun membrane makes the designed device particularly suitable for the treatment of injuries not surgically mendable due to their anatomical location (e.g., pelvic fractures) and the common frailty context of the patient.

In that light, in this study the authors proved the successful development of a *one-step* strategy to simultaneously crosslink and functionalize the electrospun collagen membrane, with the preservation of the biomimetic nanostructure imparted by the electrospinning process and ICOS-Fc biological functionality.

**Figure 1:**
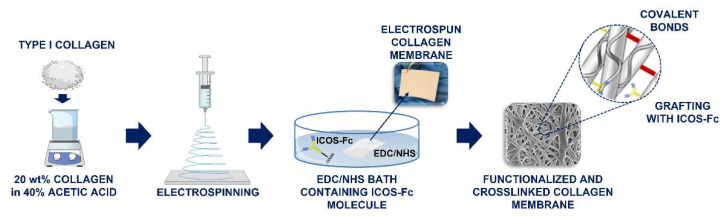
Schematic outline of the electrospun crosslinked collagen membranes functionalized with ICOS-Fc molecules.

At first, the retention of the collagen structural integrity upon ESP process was assessed, since the protein degradation is a commonly reported issue when organic solvents are involved [13]. Secondly, an EDC/NHS crosslinking strategy has been optimized to promote the simultaneous crosslinking of collagen molecules and the covalent attachment of ICOS-Fc (through the carboxylic groups exposed on Fc residue) to free amine moieties exposed by the protein chains. The following scheme (Figure 1) outlines the multistep procedure applied to obtain crosslinked biofunctionalized collagen membranes.

Finally, in vitro biological assays aimed at proving the effective binding of ICOS-Fc onto the electrospun membrane and the overall biocompatibility of the final device. Moreover, as proof of retained functionality for the anchored ICOS-Fc, the ability to inhibit the motility of human osteosarcoma cells expressing ICOSL (ICOS-Fc surface receptor) has been investigated, in analogy to the studies performed using the free form of the functional molecule [21].

To the best of the authors’ knowledge, this strategy is not yet reported in the literature and, therefore, represents a valuable contribution to the field of BTE.

## 2. Materials and Methods

### 2.1. Materials

Type I collagen was extracted from rat tail (N-COL) by NOVAICOS. ICOS-Fc, ICOSL (Sino-Biological, Inc., Beijing, China) and anti-ICOS-Fc antibody (C398.4A) were produced and provided by NOVAICOS. The following materials were purchased from Sigma Aldrich: glacial acetic acid (AA), *N*-(3-Dimethylaminopropyl)-*N*′-ethylcarbodiimide hydrochloride (EDC), 1-Hydroxy-2,5-pyrrolidinedione (NHS), pure ethanol (EtOH), and double distilled water (ddH_2_O).

#### 2.1.1. Collagen Extraction and Preparation of ESP Solution

Type I collagen was extracted from rat tail tendons (N-COL), using a protocol developed in-house, in line with the one reported by Rajan et al. [23]. Briefly, the collagen fibres were dissected into small portions and dried under a biological hood. The dried fibres were weighted and transferred in 0.2% acetic acid (0.2%AA) creating a stock solution. The collagen was stirred at 4 °C for 3 days, and then triturated using a standard hand blender, in an ice bath to prevent overheating. The obtained homogenized mixture was centrifuged at 3500 rpm, at 4 °C for 45 min, then filtered and stored at 4 °C. Prior to use, the collagen was aliquoted in 50 mL tubes, and frozen at −20 °C for 24 h. The samples were then lyophilized with a Lyovapor L-200 freeze-dryer (Büchi, Switzerland) under vacuum (<0.1 mbar) for 72 h. 1 g of N-COL was added to 5 mL of a solution of acetic acid (40% *v*/*v*) in ddH_2_O (40%AA), to achieve a final concentration of N-COL 20% *w*/*w* (named hereafter 20N-COL). The solution was left to stir overnight, at room temperature, to ensure full dissolution of the collagen.

#### 2.1.2. Production of ICOS-Fc Recombinant Molecule

Based on the work of Di Niro et al. [24], the extracellular portion of human ICOS was cloned as a fusion protein to the human IgG1 Fc region, generating the ICOS-Fc recombinant protein. After stable transfection, cells were able to express and secrete ICOS-Fc in the culture supernatant as a soluble protein. The human ICOS-Fc was harvested and purified from the supernatant via protein G affinity chromatography.

### 2.2. Assessment of the Structural Integrity of Extracted Collagen before and after ESP

#### 2.2.1. Circular Dichroism Analysis (CD)

The CD analysis was performed on N-COL as provided and after being dissolved in 40%AA (20N-COL). The 20N-COL sample was prepared as in Section 2.1.1, transferred into a 5 mL Eppendorf, frozen at −20 °C overnight and then lyophilized under vacuum (<0.1 mbar) for 24 h. Then, 5 mg of each sample (N-COL and 20N-COL) was added to 5 mL of ddH_2_O under stirring at room temperature, attaining a concentration of 1 mg/mL. To avoid the signal saturation (absorbance higher than 1.0), a calibration step was performed for each sample where collagen was diluted up to a concentration of 0.1 mg/mL. The CD analysis was performed on diluted samples (0.1 mg/mL), using a JASCO J-815 Circular Dichroism Spectropolarimeter, equipped with a Xe arc lamp, to record data in the far-UV spectral range. A total number of 3 scans were recorded for each sample at 50 nm/sec scanning rate and at 20 °C to obtain the final averaged CD spectra, and the data analysed with the Spectra Analysis software, purchased by JASCO. All the tests were performed using a quartz circular cuvette with a path length of 0.1 mm in the 180–260 nm wavelength range. Correction of spectra were performed considering the correspondent solvent medium as baseline (ddH_2_O).

#### 2.2.2. Sodium Dodecyl Sulphate–Polyacrylamide Gel Electrophoresis (SDS-PAGE)

The SDS-PAGE analysis was performed on lyophilized samples of N-COL and 20N-COL (prepared as in Section 2.1.1). The solutions for SDS-PAGE were prepared through the in-house standard procedure for this assay. Briefly, the control samples, N-COL as extracted (2 mg/mL) and type I collagen from rat tail, Roche (the commercial reference sample, 3 mg/mL), were dissolved in 0.2%AA to ensure preservation of N-COL whilst attaining full dissolution. Solutions reached a final concentration of 2 mg/mL, deemed ideal to obtain a reliable result from the SDS-PAGE analysis. The dissolution of 20N-COL samples proved to be difficult, meaning the aforementioned procedure had to be adapted and optimized. In details, 100 mg of lyophilized 20N-COL were added to 50 mL of 0.2%AA and the solution left stirring overnight, at 4 °C. The resultant solution was homogenized with a hand blender and left stirring for 2 additional days, at 4 °C. To ensure the final solution possessed the amount of protein needed for the analysis, a Bicinchoninic Acid Protein Assay (BCA) was performed. Since the BCA assay demands a fully homogeneous solution, free of undissolved material and possible agglomerates, the solution was re-blended and filtered through a 100 µm strainer. At this stage, the resulting sample was quantified using BCA, revealing a final concentration of 1.7 mg/mL.

For the SDS-PAGE analysis, 2 and 4 μg of each preparation (N-COL, 20N-COL, and Roche collagen) were resuspended into a loading buffer, heated for 5 min at 95 °C, and then loaded into the gel wells to perform the run. To visualize the bands, a Coomassie gel staining solution was prepared by mixing 0.1 Coomassie brilliant blue R-250 (Sigma-Aldrich, Burlington, MA, USA) in a solution of 45% methanol and 10%AA. This solution was added to the protein bands for 30 min, and then replaced by a Coomassie gel de-staining solution consisting in 10%AA and 10% methanol. The de-staining solution was left to develop overnight, until the bands were clearly detectable. Pre-stained protein size markers (Thermo-Fisher, Waltham, MA, USA) were used to estimate the apparent size of the collagen subunits.

### 2.3. Processing and Functionalization of the Electrospun Collagen Membranes

#### 2.3.1. ESP of 20N-COL

The 20N-COL solution was prepared using the method described previously in Section 2.1.1, then transferred to a 10 mL syringe and electrospun for 3 h onto a plate collector covered by aluminium foil, at flow rate of 300 µL/h, a voltage of 22 kV and a working distance of 12 cm. The obtained membranes (20N-COL/ESP) were left to air dry overnight, at room temperature.

#### 2.3.2. Crosslinking and Functionalization of the Electrospun Membranes

The electrospun collagen-based scaffolds were cut into 1 × 1 cm squares (n = 3), placed in 12-well plates, and incubated at 4 °C overnight. Then, 10 mM of EDC and 5 mM of NHS were added to pure EtOH (previously incubated at 4 °C) and stirred for 5 min. Then, 2 mL of the resultant crosslinking solution was added to each well, followed by the addition of ICOS-Fc at different concentrations: 50, 75, and 100 µg/mL. Samples were incubated for 8 h, at 4 °C then quickly washed 3 times with EtOH and frozen at −20 °C, overnight. Finally, the meshes were lyophilized under vacuum (<0.1 mbar) for 24 h. Crosslinked and functionalized samples will be referred as 20N-COL/ICOS-Fc. Unfunctionalized crosslinked samples were also prepared as reference and will be addressed as 20N-COL/ESP+CL.

### 2.4. Assessment of ICOS-Fc Binding and Functionality

ELISA-like assays were performed to measure the amount of residual functional ICOS-Fc in the supernatants recovered after the crosslinking/biofunctionalization reaction (i.e., the unbound ICOS-Fc), allowing to assess indirectly the successful grafting of the biomolecule and the retention of its functionality (as the ability to bind ICOSL). The molecule was added in triplicate at different concentrations, 50, 75, and 100 μg/mL, and samples incubated for 8 h, at 4 °C. The electrospun membranes were then removed and the related supernatant was collected and centrifuged at 13 000 rpm for 15 min to allow the precipitation of residual ICOS-Fc (i.e., the unbound ICOS-Fc). The EtOH was substituted with ddH_2_O for performing the ELISA-like assays.

#### 2.4.1. ELISA-like Assay with ICOSL as Capture

ELISA plates were coated by overnight incubation at 4 °C with 1 µg/mL of human ICOSL-His (Sino-Biological, Beijing, China) in Phosphate Buffer Solution (PBS) 1X, pH 7.4. After washing 5 times with 0.05% Tween-20 in PBS 1X (pH 7.4), non-specific binding was blocked by incubating in the same solution at room temperature for 1 h. Subsequently, samples were added in duplicate and incubated at 37 °C for 2 h and then washed 5 times with horseradish peroxidase (HRP)-conjugated SV5 (Thermo-Fisher) and incubated for 1 h, at room temperature. TMB (tetra-methyl-benzidine) (Merck Life Science, Darmstadt, Germany) (5 times) was added to each well and the reaction stopped by adding H_2_SO_4_ 2N (Merck Life Science). A plate reader spectrophotometer at 450 nm (Packard SpectraCount, Meriden, CT, USA) was used to analyse all the samples and the results were recorded as optical density (OD).

#### 2.4.2. ELISA-like Assay with Anti-ICOS (Clone C398.A) as Capture

The ELISA assay was also performed with anti-ICOS (clone C398.4A) as capture, which can bind and detect ICOS-Fc also if non-functional, allowing to evidence any potential denaturation occurred during the binding step. To this purpose, ELISA plates were coated with 1 µg/mL of mAb anti-ICOS clone C398.4A, by overnight incubation in PBS 1X, pH 7.4, at 4 °C. The washing and reading steps used were described in the previous Section 2.4.1.

### 2.5. Physicochemical Characterization of Electrospun Membranes

The morphology of the 20N-COL/ESP and 20NCOL/ESP+CL was analysed with Field Emission Scanning Electron Microscopy (FESEM) using a ZEISS MERLIN instrument (Carl Zeiss AG, Oberkochen, Germany). The analysis was performed on 3 samples, each one mounted onto an aluminium stub and coated with a 7 nm-thin platinum layer. The fibre and pore diameter were estimated by collecting 5 measurements from each image. Since pores were irregularly shaped, the largest distance between pore edges was considered. The final value was obtained using OriginPro2016 and presented as mean ± standard deviation (SD).

The Attenuated Total Reflection-Fourier Transform Infra-Red spectroscopy (ATR-FTIR) analysis was performed on 5 different of samples, corresponding to different phases of the process: (1) collagen as supplied (N-COL); (2) 20N-COL solution lyophilized (20N-COL); (3) electrospun membranes (20N-COL/ESP); (4) crosslinked without ICOS-Fc (20N-COL/ESP+CL); and (5) crosslinked membranes functionalized with ICOS-Fc (20N-COL/ICOS-Fc). FTIR spectra were obtained in the 4000–650 cm^−1^ range, and collected with a Bruker Equinox 55 spectrometer, equipped with MCT cryodetector, at a spectral resolution of 4 cm^−1^ and accumulation of 32 scans, by using the attenuated total reflection (ATR) mode.

The Thermogravimetric Analysis (TGA) analysis was performed with a TGA/SDTA851 (Mettler Toledo, USA) using a heating rate of 10 °C/min, within the temperature range of 23–700 °C, in air. The data were collected with STARe software and treated on OriginPro2016. The measurement of residual free amines was conducted for 20N-COL/ESP, 20N-COL/ESP+CL and 20N-COL/ICOS-Fc (50 μg/mL).

### 2.6. Measurement of Free Amine Residues with 2,4,6-Trinitrobenzene Sulfonic Acid Assay (TNBS)

Since both ICOS-Fc binding and crosslinking involves the reaction of collagen amine groups, TNBS—a UV-absorbing chromophore—was used to show the changes in the free primary amino groups of collagen, before and after crosslinking and functionalization [25]. The measurement of residual free amines was conducted for 20N-COL/ESP, 20N-COL/ESP+CL and 20N-COL/ICOS-Fc (50 μg/mL). For the analysis, 1 mL of 4% (*w*/*v*) sodium bicarbonate (NaHCO_3_, pH 8.5) was added to each sample (11 mg), followed by 1 mL of 0.5% (*w*/*v*) TNBS. The samples were placed in a dynamic shaker at 40 °C and incubated for 3 h under mild agitation. Subsequently, 3 mL of 6 M HCl were added to each sample, followed by 1 h incubation in the dynamic shaker, under mild agitation at 90 °C. Prior to the analysis, the dissolved samples were diluted in ddH_2_O (1:20), then transferred into a 5 mL cuvette for the spectrophotometry analysis. Samples were measured in triplicate and a buffer made of TNBS-only solution was used as control. The absorbance was measured in double mode with a UV-Vis-NIR spectrophotometer (Carry 5000 1.12, Agilent, Santa Clara, CA, USA) and the data obtained through the instrument’s software (Scan 3.0). The peak of interest was identified as 346 nm [26,27] on the data that was later processed in OriginPro2016.

### 2.7. Biological Assessment of ICOS-Fc Functionalized Collagen Scaffolds

The electrospun membranes for the following biological assays were prepared by ESP the 20N-COL solution onto round cover slips (15 mm diameter) for 15 min, using the process parameters described in Section 2.3.1. After ESP, samples were frozen at −20 °C and lyophilized. Subsequently, they were placed in a 24-well plate, immersed in 1 mL of crosslinking solution containing ICOS-Fc at a concentration of 50 µg/mL, then incubated for 1 h at 4 °C. The incubation time was established according to the indications provided by Ribeiro et al. which suggest it should match the amount of collagen present in each membrane [15]. Prior to cell seeding, samples were gradually rehydrated in EtOH/ddH_2_O at the following concentration (*v*/*v*): 100% EtOH, 90%, 70%, 60%, 50%, 40%, 30%, 15%, 10%, and 100% ddH_2_O.

#### 2.7.1. Cells for Biocompatibility and Migration Assays

Human osteosarcoma cell line U2OS (ICOSL positive) was obtained from the American Type Culture Collection (Manassas, VA, USA) and grown as a monolayer in DMEM (Gibco, Life Technologies, Carlsbad, CA, USA) while human osteosarcoma cell line HOS (ICOSL negative) was obtained from Sigma-Aldrich and cultured in MEM (Gibco) + 1% non-essential amino acids (Sigma-Aldrich). All media were supplemented with 10% Fetal Bovine Serum (FBS), 100 U/mL penicillin, and 100 µg/mL streptomycin (Gibco), and cells maintained at 37 °C in a 5% CO_2_ humidified atmosphere.

#### 2.7.2. Cytocompatibility of 20N-COL and 20N-COL/ICOS-Fc Membranes

Sterilisation of 20N-COL/ESP+CL and 20N-COL/ICOS-Fc samples were performed under UV-light for 30 min. Before seeding, samples were incubated in DMEM for 30 min. U2OS cells were then seeded onto the samples, plating 30 × 10^3^, 7.5 × 10^3^, and 1.5 × 10^3^ cells in 1 mL/well for 3–5–7 days, respectively, in complete DMEM medium (Gibco) and the samples incubated at 37 °C, in a 5% CO_2_ humidified atmosphere. At each time point, viable cells were evaluated by adding XTT [2,3-Bis(2-methoxy-4-nitro-5-sulfophenyl)-2H-tetrazolium-5-carbox-anilide)] reagent (Trevigen, Helgerman CT, Gaithersburg, MD, USA) for 3 h at 37 °C. A plate reader spectrophotometer at 490 nm (Packard SpectraCount) was used to read all samples and cell viability was calculated according to the following formula:

cell viability = ((absorbance of sample)/(absorbance of control (cells alone)) × 100

#### 2.7.3. Assessment of Cell Motility

Prior to cell seeding, samples were gradually rehydrated in EtOH/ddH_2_O using protocol described in Section 2.6. The cells were seeded on the collagen membranes for 30 min at 37 ° C, then, detached, counted, and used for the migration assay (2 × 10^3^ cells in 50 μL/well). To perform the Boyden chamber migration assay (BD Biosciences, Milan, Italy) cells were plated onto the apical side of 50 μg/mL Matrigel-coated filters (8.2 mm diameter and 0.5 μm pore size; Neuro Probe, Inc.; BIOMAP snc, Milan, Italy) with the addition of serum-free medium. In parallel, medium containing 20% FBS was placed in the basolateral chamber as a chemoattractant and after 6 h, cells on the apical side were wiped off with Q-tips. Methanol and crystal-violet were subsequently used to stain the cells present at the bottom of the filter and cell count was carried out with an inverted microscope. The data have been expressed as percentages and reported as mean ± SEM (n = 5) of the percentage of migration versus control migration. Five independent experiments were performed.

## 3. Results

### 3.1. Physicochemical Characterization of Collagen and Electrospun Membranes

In order to promote the successful creation of nanofibrous electrospun membranes, a highly concentrated collagen-containing solution (20% *w*/*v*) was prepared by dissolving lyophilized type I rat tail collagen (N-COL), in an aqueous solution of 40% acetic acid (40%AA). The first essential goal of this study was the assessment of the supramolecular structure of collagen to confirm its preservation upon extraction and dissolution in the acidic medium. This was investigated by performing SDS-PAGE analysis and CD on the collagen, before and after its dissolution.

**Figure 2:**
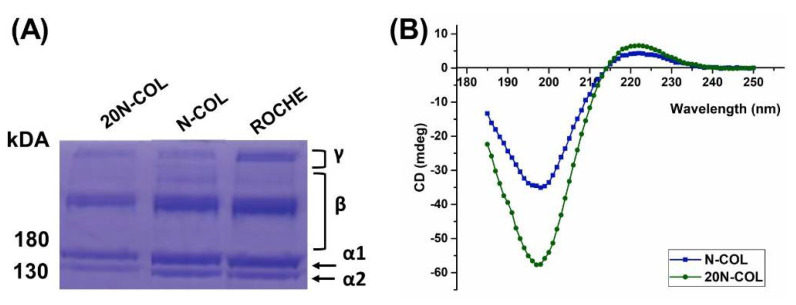
Structural integrity analysis of collagen using (**A**) SDS−PAGE and (**B**) CD.

The results from SDS-PAGE analysis performed on all collagen samples (N-COL, 20N-COL, and type I rat tail collagen from Roche) showed the presence of both α bands, as well as β and γ bands, meaning the chains were not degraded (Figure 2A) [28,29]. This confirms the identity and purity of extracted collagen in the case of N-COL, and its preservation after contact with an acidic solution (20N-COL). Moreover, the N-COL and 20N-COL samples presented the same bands of collagen produced by Roche (chosen as commercial benchmark), confirming their high quality. Both collagen samples, as extracted (N-COL) and after dissolution in acidic medium (20-NCOL), revealed very similar CD patterns (Figure 2B), characteristic of the collagen triple-helix conformation, with a weak positive band at 220 nm, and strong negative band at 198 nm [30]. Overall, this preliminary assessment demonstrated that the dissolution in 40%AA did not significantly alter the structural integrity of N-COL.

**Figure 3:**
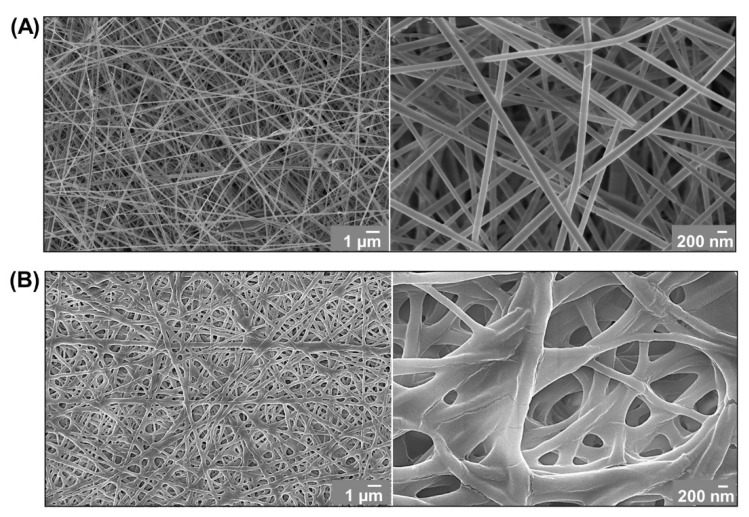
FESEM micrographs of electrospun collagen membranes before (**A**) and after crosslinking with EDC/NHS (**B**).

The obtained acidic solution of 20N-COL was subsequently used to produce nanofibrous scaffolds. To this aim, the solution was electrospun setting the applied voltage and the material flow at 22 kV and 300 μL/h, respectively, using a plate collector and keeping constant conditions of temperature and humidity. The size and morphology of the electrospun collagen fibres was assessed with FESEM (Figure 3A) revealing a homogeneous diameter, approximately 120 nm, and a cylindrical shape. The electrospun collagen membranes were subsequently chemically crosslinked using EDC/NHS in order to avoid premature dissolution in aqueous-based media and improve their mechanical strength. As visible in Figure 3B, the fibre morphology did not change significantly after crosslinking. However, the diameter increased to approximately 140 nm as a result of fibre merging, similarly to what is reported after crosslinking of collagen and other natural polymer-based electrospun structures [31,32]. The average pore diameter, after crosslinking, was reported as 1 μm ± 0.31, thus obtaining a porosity capable of promoting nutrient exchange and cell migration [33].

### 3.2. Efficiency of the Crosslinking-Biofunctionalization Strategy

To assess the ICOS-Fc grafting onto collagen fibres, ELISA-like assays were performed on the EDC/NHS solution recovered after incubation with the electrospun membranes. The assays aimed to indirectly prove ICOS-Fc grating onto collagen fibres by analysing the presence of the molecule in the supernatant (unbound ICOS-Fc), which was expected to significantly decrease due to its binding to collagen amines. A subtractive calculation method allowed an estimation of the grafted amount based on the obtained concentrations.

**Figure 4:**
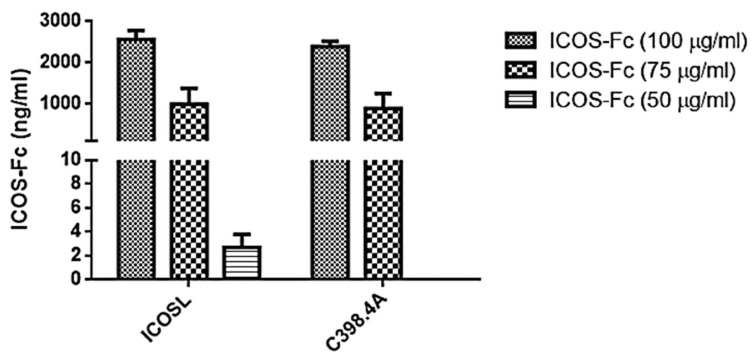
Values of ICOS-Fc obtained from ELISA assay using ICOS-L and C398.4A as capture molecules. Analysis of supernatants obtained from 20N-COL membranes incubated for 8 h in the presence of different concentrations of ICOS-Fc (100–75–50 µg/mL). The graph shows the concentration (ng/mL) as mean and standard error obtained from three independent experiments.

ELISA-like assays have been conducted by using recombinant ICOSL or anti-ICOS mAb C398.4A to capture ICOS-Fc, since the first can detect ICOS-Fc only in its functional form. At variance, anti-ICOS mAb C398.4A can detect the presence of ICOS-Fc in the supernatant regardless of the potential alterations to its functionality, which could possibly be caused by the reagents used in the crosslinking reaction. The tests performed on samples incubated for 8 h, containing different concentrations of ICOS-Fc for the functionalization step (Figure 4), revealed concentrations of ICOS-Fc between 3 and 0.002 μg/mL, using both capture tests, implying successful binding of ICOS-Fc to the collagen fibres and no significant damage of the molecule due to the crosslinking medium.

The obtained results show that the three investigated ICOS-Fc concentrations, added during the crosslinking step, allowed for the functionalization of the collagen membranes. The highest concentration of functional ICOS-Fc detected in the supernatant was ca 2.5 μg/mL, measured when the membranes were soaked into a 100 μg/mL solution, meaning that ca 97.5% of the molecules attached to the membrane. Attachment of the molecule was successfully achieved even for lower concentrations like 50 μg/mL where the amount of ICOS-Fc in the supernatant was neglectable for both capture methods, meaning almost 100% binding. This was the concentration used for the remaining tests presented in this section.

Overall, the ELISA assays suggest that ICOS-Fc molecules were effectively bound to the collagen structures with a full retention of the functionality upon the incubation.

**Figure 5:**
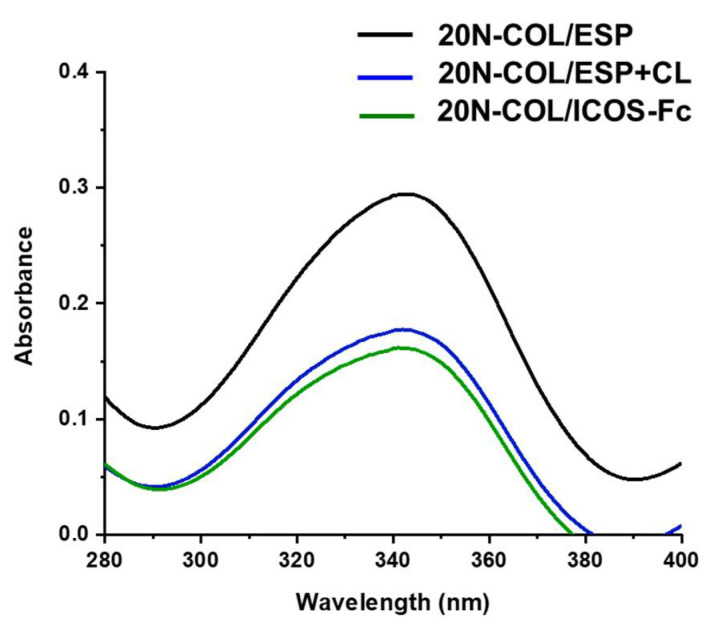
UV-Vis spectra obtained from the free amine analysis of electrospun collagen membranes, as produced (20N-COL/ESP), crosslinked without functionalization (20N-COL/ESP+CL), and crosslinked with addition of ICOS-Fc (20N-COL/ICOS).

As a confirmation of the effective protein crosslinking, and demonstration of the functionalization with ICOS-Fc, an assay to quantify free amines was performed on electrospun collagen prior to crosslinking (20N-COL/ESP), after crosslinking without ICOS-Fc (20N-COL/ESP+CL) and with ICOS-Fc (20N-COL/ICOS-Fc) (Figure 5).

The graph obtained from the UV-Vis analysis shows a significant reduction in the free amines in collagen once the sample is crosslinked (20-N-COL/ESP+CL), demonstrating that the covalent bonding between collagen chains has occurred. Moreover, a further small reduction is revealed upon ICOS-Fc functionalization (20N-COL/ICOS-Fc), suggesting the concurrent binding ICOS-Fc to the free amines, without impairing the crosslinking reaction.

**Figure 6:**
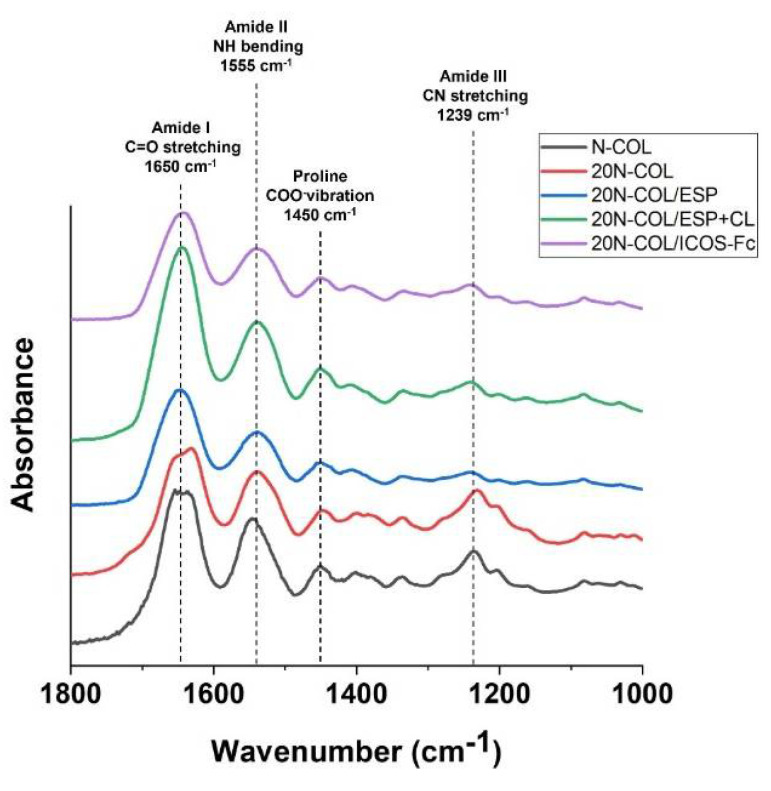
ATR-FTIR spectroscopy of samples produced with N−COL: as supplied (N−COL), after dissolution in acidic medium (20N−COL), after ESP (20N−COL/ESP), post-crosslinking with EDC/NHS (20N−COL/ESP+CL), and post-functionalization with ICOS−Fc (20N−COL/ICOS−Fc).

The ATR-FTIR analysis (Figure 6) allowed to study the tertiary structure of collagen before (N-COL) and after processing.

Based on the literature, the features of the following absorption bands were considered: amide I at 1650 cm^−1^ (C=O stretching), amide II at 1555 cm^−1^ (N-H bending), amide III at 1239 cm^−1^ (CN stretching and NH bending), and carboxylate groups of proline at 1450 cm^−1^ (COO^−^ vibration) [34,35,36].

FTIR spectra showed for all samples the bands ascribed to the amide I (1650 cm^−1^), the amide II (1555 cm^−1^), and the proline stretch (1450 cm^−1^). The amide II band did not suffer significant alterations [34,37] while the amide III band demonstrated a gradual widening when collagen is processed by electrospinning and further crosslinked and functionalized. In details, immediately after electrospinning the band is less evident, with a restoration of higher intensity upon crosslinking. These variations in the amide III band suggest a potential change in the protein orientation following the processing steps as suggested by different authors in the literature [13,38]. Overall, the spectra indicate that no significant alterations occurred to the collagen tertiary structure since all the main characteristic peaks of the protein were preserved without registering evident shifts.

**Figure 7:**
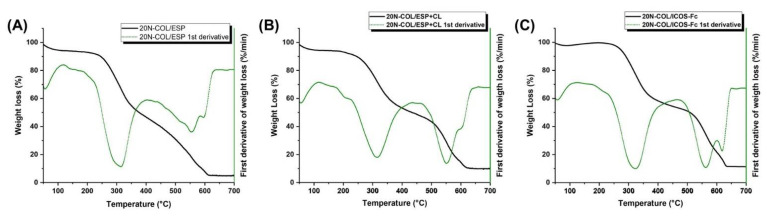
TGA analysis and first derivative of samples produced with (**A**) N-COL after ESP (20N-COL ESP), (**B**) post-crosslinking with EDC/NHS without ICOS-Fc addition, and (**C**) post-crosslinking, functionalized with ICOS-Fc.

The structural variations generated by the crosslinking process were also evaluated by TGA (Figure 7A,B). For collagen there are three ranges of temperature where the main weight losses are shown: I (50–200 °C), II (200–450 °C), and III (450–700 °C) [39,40].

The first loss relates to the desorption of physiosorbed water from collagen samples^,^ both pre- and post-crosslinking, and it is identified by the first negative peak in their derivative curves (green line). The 20N-COL/ESP (Figure 7A) presents a slightly higher percentage of weight loss as visible from the first derivative, suggesting larger amount of retained molecular water, compared to 20N-COL/ESP+CL and 20N-COL/ICOS-Fc (Figure 7B,C), which due to consumption of amine groups during the crosslinking reaction results less prone to engage the H-bonding with water molecules. The second stage, between 200 °C and 400 °C, indicates the release of water bound to collagen and degradation products of the collagen chains. The electrospun collagen before crosslinking (Figure 7A) depicts most of the weight loss between 250 and 300 °C, as clearly shown by the first derivative curve. The crosslinked collagen (Figure 7B) and the functionalized one (Figure 7C) also present a less intense loss at 300 °C compared to the non-crosslinked collagen, implying that it suffers less degradation as a result from the successful crosslinking reaction. After 400 °C, the non-crosslinked collagen showed a minor weight loss related to the degradation of the residual organic components, in contrast with the crosslinked and functionalized samples, which presented a significant additional loss around 500 °C. This indicated that the structure did not fully decompose at lower temperatures. In general, before 500 °C the non-crosslinked sample lost 68% of the initial weight, while the crosslinked and functionalized ones 55% and 53%, respectively, with a difference of more than 10%. This difference was maintained up to 600 °C, the point at which all samples had lost over 80% of the total weight.

### 3.3. Cytocompatibility of Functionalized Membranes and Influence on Osteosarcoma Motility

**Figure 8:**
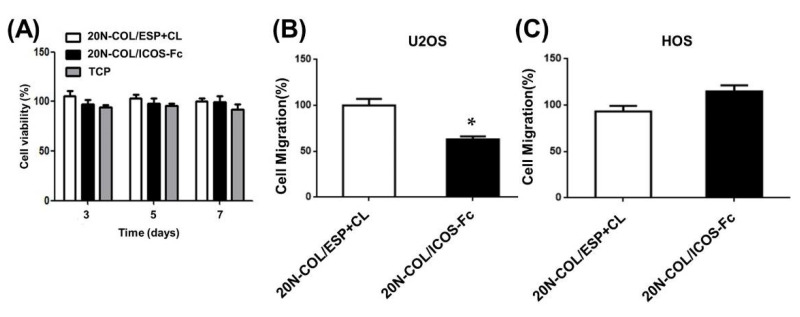
Cell viability and migration studies performed on electrospun collagen membranes, with and without ICOS-Fc. (**A**) Viability of U2OS cells when in contact with 20N-COL/ESP+CL and 20N-COL/ICOS-Fc samples, tissue culture plastic (TCP), at different timepoints: 3, 5, and 7 days. The graph shows the cell viability (%) as mean and standard error obtained from five independent experiments. (**B**) U2OS (ICOSL positive) and (**C**) HOS (ICOSL negative) cells migration after contact with collagen membranes functionalized with ICOS-Fc (20N-COL/ICOS-Fc) or not (20N-COL/ESP+CL). Data are expressed as mean ± SEM (n = 5) of the percentage of migration versus control migration in the absence of any collagen scaffold. * *p* < 0.05.

To evaluate cytotoxicity, U2OS human osteosarcoma cells were seeded on 20N-COL/ESP+CL or 20N-COL/ICOS-Fc samples. 3, 5, and 7 days after seeding, cell viability was assessed by using XTT reagent. As reported in Figure 8A, the registered values did not evidence significant differences in cell viability compared to cells seeded on tissue culture plastic (TCP) over time, meaning the membranes are not cytotoxic.

The retention of the ICOS-Fc ability to inhibit cell migration [21] was assessed through the Boyden chamber migration assay with either the ICOSL positive cell line U2OS (Figure 8B) or the ICOSL negative cell line HOS (Figure 8C). Results showed that the exposure of U2OS cells to 20N-COL/ICOS-Fc membranes inhibited their migration by 40%, compared to 20N-COL/ESP+CL membranes. This confirmed the proven biological inhibitory effect of ICOS-Fc on cell migration [22,41]. By contrast, the migration of the ICOSL negative HOS cells was not affected by the contact with 20N-COL/ICOS-Fc scaffolds, which was predictable as these cells are not responsive to its inhibitory action. No differences were found between control samples (TCP and 20N-COL/ESP+CL).

## 4. Discussion

Collagen, as one of the main constituents of bone, is a common choice as a base material for the development of scaffolds aiming at BTE. The combination of ESP with collagen can boost the degree of biomimicry of a device as it allows the creation of structures that are similar to the ECM, at both compositional and architectural level [6].

In this work, an aqueous solution of acetic acid was used as mild solvent for the dissolution of collagen in order to avoid the protein denaturation and allow the electrospinning process [16,42]. To investigate the effects of the solubilization and ESP process on collagen’s structural integrity, CD and SDS-PAGE analysis were performed (Figure 2), both confirming the presence of the triple helical structure. At the molecular level, the triple helix of collagen type I is composed of three identical α-chains (two α_1_ chains and one α_2_), each one composed of a repeating amino acid sequence of glycine-X-Y, where typically X is a proline and Y is an hydroxyproline [28,29]. The three α chains then assemble into a triple helix by coiling around each other in a rope-like fashion, forming tropocollagen. In SDS-PAGE analysis, these chains are represented in an electrophoretic profile as bands. The analysis performed on N-COL, 20N-COL, and the commercial reference Roche (Figure 2A) shows the α_1_ chains at 180 kDa and the α_2_ at 130 kDa. The representation of α_1_ and α_2_-chains together, or two α_1_-chains, is seen by the presence of the β-band, a dimer, which is seen at 250 kDa. Finally, the confirmation that the three α-chains are together, possibly arranged as a triple helix, is given by the γ-band, which was present for all samples. The data from SDS-PAGE was further confirmed by the CD analysis (Figure 2B), where the strong positive peak at 220 nm indicates the presence of a triple helix conformation [30]. Altogether these results show that the used solvent system allowed to preserve the protein structure ensuring the full retention of its bioactivity.

ATR-FTIR spectroscopy provided further insights on collagen structure upon each process step. The collected spectra (Figure 6) show that all samples presented the expected amide bands, with exception of 20N-COL/ESP which did not present the amide III signal. This was associated with a slight loss of molecular structure [36], specifically to β-sheet secondary structures, associated with a band at 1239 cm^−1^ [34]. The loss of this band can be ascribed to the fast solvent evaporation and fibre assembly occurring during ESP, which together with the dissolution in the solvent, can affect to some extent the structural conformation of the protein [36]. Similar results are reported in the studies by Sizeland et al. [10], where the results of the ATR-FTIR of electrospun membranes suggested that the alteration occurred, although the SDS-PAGE of their membranes evidenced that the α chains were intact. Their study concluded that the protein was not degraded, but the triple helices did not redevelop after ESP. The spectrum registered after crosslinking shows that the amide III band reappears, highlighting the effect of crosslinking in promoting the reconstitution and stabilization of the collagen molecular structure.

Despite the positive effects in terms of collagen stability, crosslinking treatments are often associated with loss of morphology (e.g., fibre merging and increase in fibre diameter). Therefore, minimizing the post-processing steps and optimizing the crosslinking strategy is key to ensure that the desired microstructure is preserved. In this work, this was achieved by combining the crosslinking and functionalization in a *one-step* reaction, by the simultaneous activation via EDC/NHS of the carboxylic groups exposed both by collagen and by ICOS-Fc molecule [17]. The successful outcome of the functionalization step was confirmed by ELISA-like assays which showed a reduction in ICOS-Fc in the supernatant collected from the crosslinking reaction (Figure 4). This was seen for all the three concentrations tested (50, 75, and 100 μg/mL), however, due to the high costs of ICOS-Fc (200 EUR/mg), in this instance experiments were performed with 50 μg/mL, to attain a functionalization that is both efficient and easily translated commercially. The UV-Vis analysis (Figure 5), performed on the samples exposed to 50 μg/mL of ICOS-Fc, confirmed the obtained results for crosslinking and functionalization through a significant decrease in the amount of free amines after crosslinking, and significantly lower after the grafting with ICOS-Fc.

To further investigate how crosslinking improved collagen’s stability, a TGA analysis was performed (Figure 7), showing that the process led to the increase in the denaturation temperature of ca 10 °C for crosslinked membranes. UV-Vis and TGA results clearly evidenced a high degree of collagen crosslinking, which is expected to provide enhanced biodegradation kinetics and bioactivity (angiogenesis [43], osteogenesis [44]).

The crosslinked membranes remained intact for 7 days during the cytotoxicity assay, confirming that crosslinking reaction provided a significant improvement in the overall stability and that the electrospun membranes would be able to support cell migration and proliferation.

The produced electrospun collagen fibrous membranes are expected to provide multiple biologicals and topological cues that can modulate cell adhesion, proliferation and/or differentiation and aims to provide an effective alternative strategy to the more common combination with growth factors and cells to enhance and support the process of bone formation [45,46]. In this study, with the aim to widen the exerted biological functions, the collagen-based scaffolds have been further biofunctionalized with ICOS-Fc molecule [17,21,22]. The target of the followed approach is the combination in a single multifunctional platform of several abilities to support osteoblast growth and function, whilst temporarily inducing the inhibition of osteoclasts activity through the peculiar biological properties of ICOS-Fc. The resulting multifunctional device is expected to actively contribute to the process of bone regeneration, which is key when the physiological remodelling mechanism is delayed or even altered, as in the case of elderly people or patients affected by osteoporosis.

The biological assays demonstrated that the electrospun membranes are fully cytocompatible, confirming that neither the electrospinning process nor the chemicals of the cross-linking/functionalization step have altered the overall biocompatibility.

Since the effect of free ICOS-Fc on cell migratory activity was previously reported by the authors [22] the retention of this essential biological effect was also assessed for ICOS-Fc grafted on collagen membranes by using the Boyden chamber migration assay, both with ICOSL-positive and ICOSL-negative cell lines, as a *proof of concept* of the efficacy of the developed approach. The results of this assay evidenced a 40% inhibition of U2OS (ICOSL-positive cells) migratory activity after only 30 min of contact between cells and ICOS-Fc functionalized membranes. This finding fully confirmed the retention of the inhibitory effect of ICOS-Fc anchored to electrospun membranes, in analogy to its free form [21,22] and when grafted onto the surface of bioactive glass particles [17]. ICOSL-negative cells (HOS) were used as a control, and exhibited no changes in their migration behaviour, attesting once more the specificity of the inhibitory effect derived from the ICOS:ICOSL binding at the collagen surface.

Further in vitro studies will aim at exploring the action of the functionalized membranes on osteoclast-precursor cells to evaluate the inhibitory effect on cell differentiation and the downregulation of osteoclast differentiation genes.

## 5. Conclusions

The work presented reports the successful development of *one step* strategy to achieve simultaneously the crosslinking and biofunctionalization of electrospun collagen membranes. The ICOS-Fc molecule, chosen for its ability to reversibly inhibit osteoclast function by binding its ligand ICOSL, has been effectively grafted on the collagen membranes to impart multifunctional biological effects and thus promoting the remodelling process of bone tissue in compromised clinical situations.

The stability of the ICOS-Fc binding was assessed by an *in house*-developed ELISA-like assay, revealing a functionalization yield higher than 95%, with a full retention of functionality by the grafted biomolecules.

After 7 days in culture, cells showed high viability regardless of the presence of ICOS-Fc, suggesting that neither the crosslinking nor the functionalization have altered the cytocompatibility of the electrospun collagen scaffolds. The contact of U2OS, chosen as ICOSL-positive cells, with the collagen membranes exposing ICOS-Fc at their surface resulted in the inhibition of cell migration, confirming once more the successful binding to the collagen fibres and the retention of ICOS-Fc biological properties (*proof of efficacy*). In contrast, the ICOSL-negative cell line (HOS) did not show inhibitory effects, confirming the specificity of the ICOS-Fc effect that was mediated by binding to ICOSL.

By inhibiting the migration of ICOSL-positive cells, e.g., osteoclasts, the developed membranes can play an active role on bone remodelling, placing this strategy in the category of future therapies based on cell behaviour modulation to achieve improved regeneration.

The results of this study highlight the high potential of the developed multifunctional platform (i.e., osteoconductive, pro-osteogenic, anti-clastogenic) for treating delayed bone healing and pave the way to further in vivo studies to implement minimally invasive clinical solutions (e.g., injection via cannulated instruments at the fracture site).

## Data Availability

The data presented in this study are openly available in ZENODO at 10.5281/zenodo.7063972, https://doi.org/10.5281/zenodo.7063972.
